# Early 1900s Detection of *Batrachochytrium dendrobatidis* in Korean Amphibians

**DOI:** 10.1371/journal.pone.0115656

**Published:** 2015-03-04

**Authors:** Jonathan J. Fong, Tina L. Cheng, Arnaud Bataille, Allan P. Pessier, Bruce Waldman, Vance T. Vredenburg

**Affiliations:** 1 School of Biological Sciences, Seoul National University, Seoul, South Korea; 2 Department of Biology, San Francisco State University, San Francisco, California, United States of America; 3 Department of Ecology & Evolutionary Biology, University of California Santa Cruz, Santa Cruz, California, United States of America; 4 Wildlife Disease Laboratories, Institute for Conservation Research, San Diego Zoo Global, San Diego, California, United States of America; Clemson University, UNITED STATES

## Abstract

The pathogenic fungus *Batrachochytrium dendrobatidis* (*Bd*) is a major conservation concern because of its role in decimating amphibian populations worldwide. We used quantitative PCR to screen 244 museum specimens from the Korean Peninsula, collected between 1911 and 2004, for the presence of *Bd* to gain insight into its history in Asia. Three specimens of *Rugosa emeljanovi* (previously *Rana* or *Glandirana rugosa*), collected in 1911 from Wonsan, North Korea, tested positive for *Bd*. Histology of these positive specimens revealed mild hyperkeratosis – a non-specific host response commonly found in *Bd*-infected frogs – but no *Bd* zoospores or zoosporangia. Our results indicate that *Bd* was present in Korea more than 100 years ago, consistent with hypotheses suggesting that Korean amphibians may be infected by endemic Asian *Bd* strains.

## Introduction

The pathogenic fungus *Batrachochytrium dendrobatidis* (*Bd*) is linked to declines or extinctions of more than 200 amphibian species worldwide [1–3]. While *Bd* is widespread in Asia [4–7], no epizootic events have been reported. One potential explanation is that Asia is an origin of the global pandemic *Bd* lineage [5,8] (but see [9,10] for alternative hypotheses).

*Bd* is present throughout Asia [5–7,11–16]. Unique genotypes in Japan [5,7], China [7,11], Korea [7], and India [16,17], as well as high prevalence and low infection intensity [1,18] in Korea [3,7,19,20], support the hypothesis that some *Bd* strains are endemic to Asia. To fully understand *Bd* in Asia so that effective conservation plans can be developed, a temporal view must be included. To date, four studies have examined historical specimens in Asia [21–26], with detection in 1933 from China [25,26]. In our study, we use quantitative PCR (qPCR) and histology to screen historical amphibian specimens from Korea for the presence of *Bd*. These results provide a temporal perspective—going back a century—on *Bd* prevalence in Korea.

## Material and Methods

**Fig 1:**
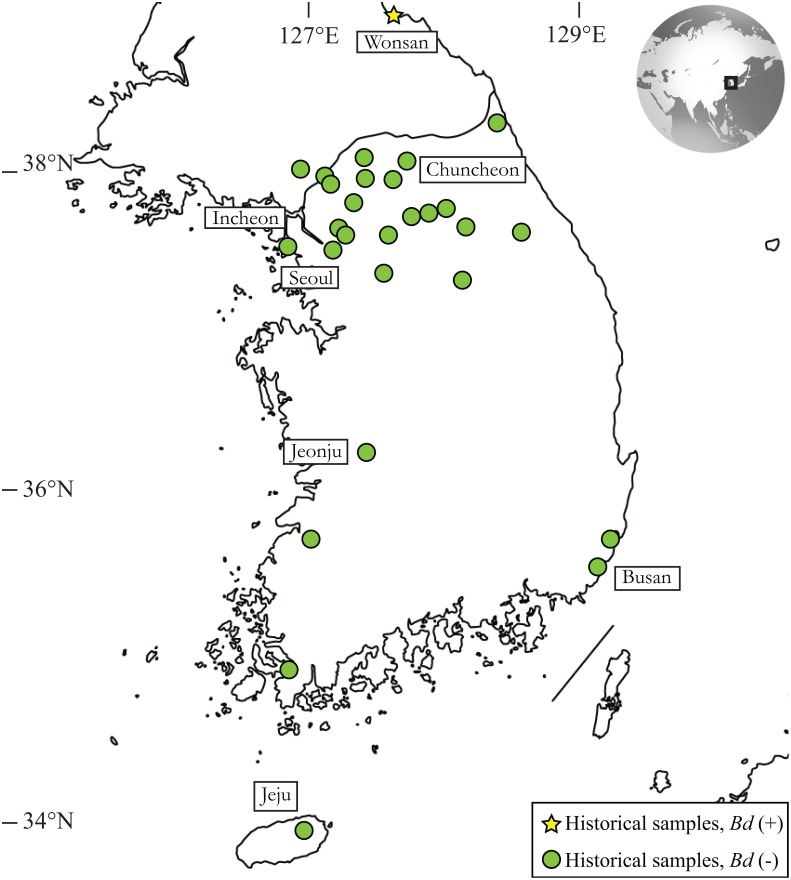
Sampling of historical specimens across the Korean Peninsula used in this study. Specimens were collected between 1911 and 2004. Three specimens of *Rugosa emeljanovi* (previously *Rana* or *Glandirana rugosa*), collected from Wonsan, North Korea in 1911, tested qPCR positive for *Batrachochytrium dendrobatidis* (*Bd*) infection.

We accessed 244 historical specimens from the Museum of Vertebrate Zoology at the University of California, Berkeley (MVZ) and the California Academy of Sciences (CAS) (S1 Table). We standardize scientific names to follow the taxonomy of AmphibiaWeb (www.amphibiaweb.org). Specimens comprise 13 of 17 species native to the Korean Peninsula and were collected between 1911 and 2004 (Table 1). We used contemporary data [6,7,14,15] (Fig. 1, Table 1) to inform our interpretation of historical data. We followed a non-invasive sampling method to detect *Bd* by the polymerase chain reaction (PCR) [27]. While specimens at the MVZ are susceptible to cross contamination because a jar of specimens of one species may represent several collecting trips to the same locality, CAS specimens are less so because they are stored in jars separated by species, locality, and collecting trip.

**Table 1:** Comparison of modern and historical specimen data.

Species	Modern samples				Historical samples					
	*Bd* +	Total	% *Bd* +	ZE	1910–1929	1930–1949	1950–1969	1970–1989	1990–2004	TOTAL
*Bombina orientalis*	62	373	16.6%	13.85	0	2	17	0	0	**19**
*Bufo gargarizans*	3	93	3.2%	20.00	0	0	2	0	0	**2**
*Bufo stejnegeri*	2	36	5.6%	-	0	0	0	0	0	**0**
*Hyla japonica*	62	420	14.8%	28.07	0	0	48	0	0	**48**
*Hyla suweonensis*	0	4	0%	-	0	0	0	0	0	**0**
*Hynobius leechii*	59	123	48.0%	1.50	0	0	0	20	18	**38**
*Hynobius quelpaertenses*	46	117	39.3%	-	0	0	0	0	0	**0**
*Hynobius yangii*	0	6	0%	-	0	0	0	0	1	**1**
*Kaloula borealis*	0	20	0%	-	0	0	4	0	0	**4**
*Karsenia koreana*	3	9	33.3%	2.67	0	0	0	0	5	**5**
*Onychodactylus fischeri*	0	22	0%	-	0	0	0	1	5	**6**
*Pelophylax chosenicus*	1	13	7.7%	-	0	0	1	0	0	**1**
*Pelophylax nigromaculatus*	5	168	3.0%	-	0	0	13	2	0	**15**
*Rana (Lithobates) catesbeiana*	28	102	27.5%	305.36	0	0	0	0	0	**0**
*Rana coreana*	11	60	18.3%	-	0	0	18	0	0	**18**
*Rana dybowskii*	46	255	18.1%	5.17	0	0	0	5	0	**5**
*Rana huarenensis*	2	14	14.3%	-	0	0	0	0	0	**0**
*Rana sp*.	0	1	0%	-	0	0	33	0	0	**33**
*Rugosa emeljanovi*	19	98	19.4%	14.12	7*	0	42	0	0	**49**
**TOTAL**	**349**	**1,934**	**18.0%**	**61.18**	**7**	**2**	**178**	**28**	**29**	**244**

Historical samples grouped into ~20 year categories. Of all historical samples tested, only three samples of *Rugosa emeljanovi* (previously *Rana* or *Glandirana rugosa*) were positive for *Batrachochytrium dendrobatidis* (*Bd*). ZE denotes the estimated *Bd* zoospore genomic equivalents on each swab. Data for modern samples are compiled from previous publications [6,7,14,15].

* Three of these historical samples tested positive for *Bd*.

Each specimen was rinsed with 70% EtOH and then swabbed (MW113, Medical Wire and Equipment, Corsham, UK) 30 times across its dorsal and ventral surfaces. Swabs were stored dry in 1.5 mL microcentrifuge tubes at 4° C until processed. Prior to extraction, swabs were dried in a Spin Vac (Savant Instruments, Farmingdale, NY, USA) to remove EtOH. Extraction was performed using 40 μL of Prepman Ultra (Applied Biosystems, Carlsbad, CA, USA) [19,20] and diluted 1:10 with 0.25 × TE buffer. We analyzed each sample in duplicate using 5 μL of the diluted DNA extract. Universal DNA standards from the global pandemic lineage (provided by A.S. Hyatt) were included to calibrate the qPCR (0.1, 1.0, 10, and 100 zoospore equivalents per reaction). Negative controls were included during extraction and qPCR to detect contamination. Samples were run on an Applied Biosystems 7300 Real-Time PCR thermocycler. A specimen was considered *Bd*-positive if one or both qPCR replicates were positive. We calculated the number of zoospores in terms of Z_swab_ (i.e., estimated *Bd* zoospore genomic equivalents on each swab) [1,3] by multiplying qPCR results by 80 to account for sample dilution (40 μL Prepman × 10 dilution / 5 μL for reaction = 80).

Three additional tests were performed to check *Bd*-positive samples. First, we ran two more qPCR replicates from the original DNA extract. Next, the positive specimen and all other specimens in the same jar were swabbed again and processed using the same qPCR protocol as above (duplicate runs per sample). Lastly, skin samples of qPCR positive specimens were screened for *Bd* using histological methods [4]. Histology was conducted at the Wildlife Disease Laboratories at San Diego Zoo (by AP) on full-thickness skin (4 × 4 mm) excised from the ventral pelvic area (n = 2) and webbing between rear digits (n = 4) from each specimen. Past studies showed these areas are likely to harbor *Bd* infection [8]. Samples were routinely processed for paraffin embedding, sectioned at 5 to 6 μm, and stained with hematoxylin and eosin [9]. In all, 120 serial sections were examined from each skin sample for the presence of *Bd* thalli, zoosporangia, and associated lesions.

## Results

From qPCR assays, 241 samples scored negative and three samples (CAS32672, CAS33676, CAS33678) positive with low levels of *Bd* amplification (Table 2). Four to six qPCR reactions (2–4 from first swab, 2 from second swab) were run for each positive sample. For the first round of qPCR, a single reaction of sample CAS32672 was positive (Z_swab_ = 0.008). The second round of qPCR from the same swab extract yielded the same result, with a single positive out of two reactions (Z_swab_ = 0.385). From qPCR of a new swab extraction, one of two reactions was positive (Z_swab_ = 0.772). Of re-swabbed specimens from the same jar (same species, same collecting trip), two additional positive samples were discovered: CAS33676 (Z_swab_ = 0.242) and CAS33678 (Z_swab_ = 1.016). Attempts to sequence these PCR products were unsuccessful, probably due to the low DNA quantity. Histological analysis of skin from the three specimens did not reveal the presence of *Bd* thalli or zoosporangia, but showed evidence of mild parakeratotic hyperkeratosis.

**Table 2:** Quantitiative PCR (qPCR) results of frog specimens that screened positive for *Batrachochytrium dendrobatidis* (*Bd*) infection.

Specimen #	Swab 1				Swab 2	
	Reaction 1	Reaction 2	Reaction 3	Reaction 4	Reaction 1	Reaction 2
CAS32672	0	0.008	0.385	0	0	0.772
CAS33676	0	0	-	-	0.242	0
CAS33678	0	0	-	-	0	1.016

Values of positive qPCR reactions are calculated as Z_swab_ (estimated *Bd* zoospore genomic equivalents on each swab) by multiplying qPCR results by 80 to account for sample dilution (40 μL Prepman × 10 dilution / 5 μL for reaction = 80).

## Discussion

We discovered three *Bd*-positive individuals out of 244 historical specimens (1.2%) from the Korean Peninsula. All positives were of *Rugosa emeljanovi* (previously *Rana* or *Glandirana rugosa*) from Wonsan, North Korea collected in 1911. Infection intensities ranged between 0.008 and 1.016 Z_swab_ (Table 2). Histological inspection of the positive samples revealed mild hyperkeratosis, which would be expected if subjects had been infected by *Bd*. These results demonstrate that *Bd* was present in Korea in the early 1900s, consistent with hypotheses suggesting the existence of endemic *Bd* strains in Asia [7].

Recent surveys in South Korea found a *Bd* prevalence of 18% [7] and average infection intensities for species ranging between 305.36 zoospore equivalents in the introduced American bullfrog (*Rana* [*Lithobates*] *catesbeiana*) and 1.50 zoospore equivalents in the Korean salamander (*Hynobius leechii*) (Table 1). Modern samples of *Rugosa emeljanovi* showed a *Bd* prevalence of 19.4% and an infection intensity of 14.12 zoospore equivalents (Table 1), values greater than historical samples in our study (prevalence = 6.1%, infection intensity = 0.008–1.016 Z_swab_; Table 2). The inconsistencies revealed when comparing historical and modern data may be attributable to three factors: 1) increased *Bd* prevalence in recent times by either an endemic strain or emergence of a new strain (e.g. [28]), 2) reduced detection in historical samples due to degraded DNA or the presence of PCR inhibitors [29], or 3) sampling error associated with the availability of historical specimens. Our data cannot differentiate among these possibilities. Although we know that *Bd* was present in Korea in 1911, we do not know its status between 1911 and present time. Further study involving time-calibrated genetic analyses and additional historical specimens is needed to determine how *Bd* spread through Korea over time.

We minimized the possibility of false positives for the three specimens collected in 1911 by running replicate tests on each sample using two different methods—qPCR and histology. All tests consistently yielded the same result—low *Bd* infection loads in the three specimens. The robustness of our conclusions is boosted by a recent study that found low probabilities of false positives due to contamination using the same qPCR assay that we used [18]. Although uncertainty exists as to how the museum specimens were handled in the past, we undertook precautions to minimize sample contamination by continually sterilizing equipment and the workspace. Additionally, specimens from CAS were stored separately by species, locality, and collecting trip, further reducing the possibility of cross-contamination. In the jar of the three positive samples, four other frogs consistently yielded negative results for *Bd* in all trials.

For qPCR, positive and negative controls were run throughout the procedure, and neither produced results that would indicate a failed reaction or contamination. One concern is the DNA standards used for qPCR (a requirement to calibrate DNA quantification) are a source of contamination. We expect contamination of samples by DNA standards to be random. However, repeated runs of positive samples consistently gave positive results. This PCR assay has been applied extensively since 2004 without any reports of false positives [3,19,20].

Difficulties of extracting and amplifying DNA from preserved specimens, especially those exposed to formalin, have been well documented [21,23] (but see [18]). There is greater success with ethanol-preserved specimens and PCR success appears negatively correlated with exposure time to formalin [30]. Therefore, the preservation history of a specimen should be considered when making conclusions using such data. The three positive samples and four negative samples stored in the same jar were collected by Joseph C. Thompson (also known as Victor Kuhne), preserved in ethanol and, to the best of curators’ knowledge, never exposed to formalin (J. Vindum, pers. comm.). The known preservation history of the three positive samples gives us greater confidence that they are true positives.

Histological analyses of the three *Bd*-positive specimens showed mild hyperkeratosis—a thickened stratum corneum. Hyperkeratosis is a non-specific host response seen in a variety of skin diseases, but associated with *Bd* infection [4]. Although no *Bd* organisms were found, histological examination of the skin is known to be an insensitive method for detection of *Bd*, especially with low-level or subclinical infections [19,31]. Evidence of *Bd* is often found in the stratum corneum and sloughed skin, so swabbing itself may have physically removed zoosporangia and destroyed histological evidence of positive infection. Histological results are consistent with qPCR results that detected low zoospore counts. Similarly, contemporary samples of native Korean amphibian species reveal low levels of *Bd* infection, with an average of 1.50 to 28.07 zoospore equivalents per species (Table 1).

Our results need to be interpreted within an historical anthropogenic context. Korea was relatively isolated from other countries until the late 1800s, when several international treaties opened ports to trade. Wonsan, the site of the three positive specimens, was opened in 1880 to trade with Japan, Russia, China, Germany, and Great Britain [32,33]. The *Bd-*positive samples were collected in 1911, which raises the possibility that *Bd* was introduced into Korea during the late 1800s or early 1900s from one of the trade partner countries. Japan and China are the most likely sources, as trade with these countries was the highest [32]. We cannot determine whether *Bd* detected in our study is native or introduced, but if introduced, its likely origin is still Asia.

Two major hypotheses have been proposed to explain the pattern of *Bd* in amphibian populations: 1) *Bd* is a novel pathogen that infects and kills naïve hosts [28], and 2) *Bd* is an endemic pathogen that infects but does not kill hosts in its native range because of a stable pathogen-host equilibrium [1]. The evolutionary history of *Bd* may be complex [10], with both endemic and novel strains existing in a region. Amphibian hosts exposed to *Bd* may acquire resistance [34], while *Bd*-naïve hosts may be more susceptible to the invasive fungus [35,36]. Understanding the geographic origins of *Bd* is thus important in interpreting the effects on hosts and in determining the best way to conserve threatened species. Worldwide, the earliest records of *Bd* are 1894 from Brazil [17], 1933 from Cameroon [37] and China [26], 1934 from Kenya [38], and 1938 from South Africa [39]. Our study pushes the presence of *Bd* in Asia back to 1911. All these studies point toward longer, potentially independent histories of *Bd* in some parts of the world. Continued testing of historical specimens coupled with repeated surveys in present-day populations are needed to give a perspective on the dynamic pathogen-host relationship and the differing effects of endemic and introduced *Bd* strains.

## Supporting Information

S1 Table Data of historical amphibian samples used in this study. Shaded cells for latitude/longitude indicate that data have been added or modified based on detailed verbatim locality data. Taxonomy was changed to follow AmphibiaWeb (www.amphibiaweb.org). Under preservation type, the preservatives used for specimen preparation followed by that used for storage are given. Preservation method of specimens was assumed to be formalin/EtOH unless we had information stating otherwise. CAS = California Academy of Sciences, MVZ = Museum of Vertebrate Zoology.
(DOCX)
