# Supplementary material for: Early 1900s Detection of Batrachochytrium dendrobatidis in Korean Amphibians
Source: PLoS One. 2015 Mar 4;10(3):e0115656. doi: 10.1371/journal.pone.0115656 (PMC4349589; doi:10.1371/journal.pone.0115656)
Supplement: S1 Table — Shaded cells for latitude/longitude indicate that data have been added or modified based on detailed verbatim locality data. Taxonomy was changed to follow AmphibiaWeb (www.amphibiaweb.org). Under preservation type, the preservatives used for specimen preparation followed by that used for storage are given. Preservation method of specimens was assumed to be formalin/EtOH unless we had information stating otherwise. CAS = California Academy of Sciences, MVZ = Museum of Vertebrate Zoology. (DOCX) [file pone.0115656.s001.docx]

**Table S1**: Data of historical amphibian samples used in this study. Shaded cells for latitude/longitude indicate that data have been added or modified based on detailed verbatim locality data. Taxonomy was changed to follow AmphibiaWeb (www.amphibiaweb.org). Under preservation type, information represents (fluid used for specimen preparation/fluid used for storage). Preservation method of specimens was assumed to be Formalin/EtOH unless we had information stating otherwise. CAS=California Academy of Sciences, MVZ=Museum of Vertebrate Zoology.

| **SPECIES** | **MUSEUM** | **ID NUMBER** | **COLLECTION YEAR** | **PRESERVATION TYPE** | **LATITUDE (°N)** | **LONGITUDE (°E)** |
| --- | --- | --- | --- | --- | --- | --- |
| *Bombina orientalis* | CAS | 10062 | 1930 | Formalin/EtOH | 37.96876 | 126.5658 |
| *Bombina orientalis* | CAS | 10063 | 1930 | Formalin/EtOH | 37.96876 | 126.5658 |
| *Bombina orientalis* | CAS | 87088 | 1952 | Formalin/EtOH | 37.9862 | 127.35557 |
| *Bombina orientalis* | CAS | 87089 | 1952 | Formalin/EtOH | 37.9862 | 127.35557 |
| *Bombina orientalis* | CAS | 87090 | 1952 | Formalin/EtOH | 37.9862 | 127.35557 |
| *Bombina orientalis* | CAS | 87091 | 1951 | Formalin/EtOH | 37.83154 | 127.50988 |
| *Bombina orientalis* | CAS | 87092 | 1951 | Formalin/EtOH | n/a | n/a |
| *Bombina orientalis* | MVZ | 61818 | 1954 | Formalin/EtOH | 37.5667 | 127 |
| *Bombina orientalis* | MVZ | 66625 | 1954 | Formalin/EtOH | 37.887 | 127.733 |
| *Bombina orientalis* | MVZ | 66626 | 1954 | Formalin/EtOH | 37.887 | 127.733 |
| *Bombina orientalis* | MVZ | 66627 | 1954 | Formalin/EtOH | 37.887 | 127.733 |
| *Bombina orientalis* | MVZ | 66628 | 1954 | Formalin/EtOH | 37.887 | 127.733 |
| *Bombina orientalis* | MVZ | 66629 | 1954 | Formalin/EtOH | 37.887 | 127.733 |
| *Bombina orientalis* | MVZ | 66630 | 1954 | Formalin/EtOH | 37.887 | 127.733 |
| *Bombina orientalis* | MVZ | 66631 | 1954 | Formalin/EtOH | 37.887 | 127.733 |
| *Bombina orientalis* | MVZ | 66632 | 1954 | Formalin/EtOH | 37.887 | 127.733 |
| *Bombina orientalis* | MVZ | 66633 | 1954 | Formalin/EtOH | 37.887 | 127.733 |
| *Bombina orientalis* | MVZ | 66634 | 1954 | Formalin/EtOH | 37.887 | 127.733 |
| *Bombina orientalis* | MVZ | 66635 | 1954 | Formalin/EtOH | 37.887 | 127.733 |
| *Bufo gargarizans* | MVZ | 61819 | 1954 | Formalin/EtOH | 37.44 | 126.92 |
| *Bufo gargarizans* | MVZ | 66636 | 1954 | Formalin/EtOH | 37.887 | 127.733 |
| *Hyla japonica* | CAS | 86976 | 1952 | Formalin/EtOH | 37.83241 | 126.81845 |
| *Hyla japonica* | CAS | 86977 | 1952 | Formalin/EtOH | 37.83241 | 126.81845 |
| *Hyla japonica* | CAS | 86978 | 1952 | Formalin/EtOH | 37.83241 | 126.81845 |
| *Hyla japonica* | CAS | 86979 | 1952 | Formalin/EtOH | 37.83241 | 126.81845 |
| *Hyla japonica* | CAS | 86980 | 1952 | Formalin/EtOH | 37.83241 | 126.81845 |
| *Hyla japonica* | CAS | 86981 | 1952 | Formalin/EtOH | 37.83241 | 126.81845 |
| *Hyla japonica* | CAS | 86982 | 1952 | Formalin/EtOH | 37.83241 | 126.81845 |
| *Hyla japonica* | CAS | 86983 | 1952 | Formalin/EtOH | 37.83241 | 126.81845 |
| *Hyla japonica* | CAS | 86984 | 1952 | Formalin/EtOH | 37.83241 | 126.81845 |
| *Hyla japonica* | CAS | 86985 | 1952 | Formalin/EtOH | 37.83241 | 126.81845 |
| *Hyla japonica* | CAS | 86986 | 1952 | Formalin/EtOH | 37.83241 | 126.81845 |
| *Hyla japonica* | CAS | 86987 | 1952 | Formalin/EtOH | 37.83241 | 126.81845 |
| *Hyla japonica* | CAS | 86988 | 1952 | Formalin/EtOH | 37.83241 | 126.81845 |
| *Hyla japonica* | CAS | 86989 | 1952 | Formalin/EtOH | 37.83241 | 126.81845 |
| *Hyla japonica* | CAS | 86990 | 1952 | Formalin/EtOH | 37.83241 | 126.81845 |
| *Hyla japonica* | CAS | 86991 | 1952 | Formalin/EtOH | 37.83241 | 126.81845 |
| *Hyla japonica* | CAS | 86992 | 1952 | Formalin/EtOH | 37.83241 | 126.81845 |
| *Hyla japonica* | CAS | 86993 | 1952 | Formalin/EtOH | 37.83241 | 126.81845 |
| *Hyla japonica* | CAS | 86994 | 1952 | Formalin/EtOH | 37.83241 | 126.81845 |
| *Hyla japonica* | CAS | 86995 | 1952 | Formalin/EtOH | 37.83241 | 126.81845 |
| *Hyla japonica* | CAS | 86996 | 1952 | Formalin/EtOH | 37.83241 | 126.81845 |
| *Hyla japonica* | CAS | 86997 | 1952 | Formalin/EtOH | 37.83241 | 126.81845 |
| *Hyla japonica* | CAS | 86998 | 1952 | Formalin/EtOH | 37.83241 | 126.81845 |
| *Hyla japonica* | CAS | 86999 | 1952 | Formalin/EtOH | 37.83241 | 126.81845 |
| *Hyla japonica* | CAS | 87000 | 1952 | Formalin/EtOH | 37.83241 | 126.81845 |
| *Hyla japonica* | CAS | 87001 | 1952 | Formalin/EtOH | 37.83241 | 126.81845 |
| *Hyla japonica* | CAS | 87002 | 1952 | Formalin/EtOH | 37.83241 | 126.81845 |
| *Hyla japonica* | CAS | 87003 | 1952 | Formalin/EtOH | 37.83241 | 126.81845 |
| *Hyla japonica* | CAS | 87004 | 1952 | Formalin/EtOH | 37.83241 | 126.81845 |
| *Hyla japonica* | CAS | 87005 | 1952 | Formalin/EtOH | 37.83241 | 126.81845 |
| *Hyla japonica* | CAS | 87006 | 1952 | Formalin/EtOH | 37.83241 | 126.81845 |
| *Hyla japonica* | CAS | 87007 | 1952 | Formalin/EtOH | 37.83241 | 126.81845 |
| *Hyla japonica* | CAS | 87008 | 1952 | Formalin/EtOH | 37.83241 | 126.81845 |
| *Hyla japonica* | CAS | 87009 | 1952 | Formalin/EtOH | 37.83241 | 126.81845 |
| *Hyla japonica* | CAS | 87010 | 1952 | Formalin/EtOH | 37.83241 | 126.81845 |
| *Hyla japonica* | CAS | 87011 | 1952 | Formalin/EtOH | 37.83241 | 126.81845 |
| *Hyla japonica* | CAS | 87012 | 1952 | Formalin/EtOH | 37.83241 | 126.81845 |
| *Hyla japonica* | CAS | 87013 | 1952 | Formalin/EtOH | 37.83241 | 126.81845 |
| *Hyla japonica* | CAS | 87014 | 1952 | Formalin/EtOH | 37.83241 | 126.81845 |
| *Hyla japonica* | CAS | 87015 | 1952 | Formalin/EtOH | 37.83241 | 126.81845 |
| *Hyla japonica* | CAS | 87016 | 1952 | Formalin/EtOH | 37.83241 | 126.81845 |
| *Hyla japonica* | CAS | 87017 | 1952 | Formalin/EtOH | 37.83241 | 126.81845 |
| *Hyla japonica* | MVZ | 61820 | 1954 | Formalin/EtOH | 37.6 | 126.96 |
| *Hyla japonica* | MVZ | 66638 | 1954 | Formalin/EtOH | 37.887 | 127.733 |
| *Hyla japonica* | MVZ | 66639 | 1954 | Formalin/EtOH | 37.887 | 127.733 |
| *Hyla japonica* | MVZ | 66640 | 1954 | Formalin/EtOH | 37.887 | 127.733 |
| *Hyla japonica* | MVZ | 66641 | 1954 | Formalin/EtOH | 37.887 | 127.733 |
| *Hyla japonica* | MVZ | 66642 | 1954 | Formalin/EtOH | 37.887 | 127.733 |
| *Hynobius leechii* | MVZ | 128177 | 1976 | Formalin/EtOH | 33.41 | 126.61 |
| *Hynobius leechii* | MVZ | 128178 | 1976 | Formalin/EtOH | 33.41 | 126.61 |
| *Hynobius leechii* | MVZ | 128179 | 1976 | Formalin/EtOH | 33.41 | 126.61 |
| *Hynobius leechii* | MVZ | 128180 | 1976 | Formalin/EtOH | 33.41 | 126.61 |
| *Hynobius leechii* | MVZ | 128181 | 1976 | Formalin/EtOH | 33.41 | 126.61 |
| *Hynobius leechii* | MVZ | 128182 | 1976 | Formalin/EtOH | 37.57 | 127.54 |
| *Hynobius leechii* | MVZ | 128183 | 1976 | Formalin/EtOH | 37.57 | 127.54 |
| *Hynobius leechii* | MVZ | 128184 | 1976 | Formalin/EtOH | 37.57 | 127.54 |
| *Hynobius leechii* | MVZ | 128185 | 1976 | Formalin/EtOH | 37.57 | 127.54 |
| *Hynobius leechii* | MVZ | 128186 | 1976 | Formalin/EtOH | 37.57 | 127.54 |
| *Hynobius leechii* | MVZ | 150332 | 1977 | Formalin/EtOH | 37.25941 | 127.38643 |
| *Hynobius leechii* | MVZ | 163727 | 1979 | Formalin/EtOH | 37 | 127.92 |
| *Hynobius leechii* | MVZ | 163728 | 1979 | Formalin/EtOH | 37 | 127.92 |
| *Hynobius leechii* | MVZ | 163729 | 1979 | Formalin/EtOH | 37 | 127.92 |
| *Hynobius leechii* | MVZ | 169093 | 1979 | Formalin/EtOH | 37.39557 | 128.68037 |
| *Hynobius leechii* | MVZ | 169096 | 1979 | Formalin/EtOH | 37 | 127.92 |
| *Hynobius leechii* | MVZ | 169097 | 1979 | Formalin/EtOH | 37 | 127.92 |
| *Hynobius leechii* | MVZ | 192708 | 1979 | Formalin/EtOH | n/a | n/a |
| *Hynobius leechii* | MVZ | 192709 | 1979 | Formalin/EtOH | n/a | n/a |
| *Hynobius leechii* | MVZ | 192710 | 1979 | Formalin/EtOH | n/a | n/a |
| *Hynobius leechii* | MVZ | 230332 | 1999 | Formalin/EtOH | 37.489 | 127.491 |
| *Hynobius leechii* | MVZ | 230333 | 1999 | Formalin/EtOH | 37.489 | 127.491 |
| *Hynobius leechii* | MVZ | 230334 | 1999 | Formalin/EtOH | 37.489 | 127.491 |
| *Hynobius leechii* | MVZ | 230335 | 1999 | Formalin/EtOH | 37.489 | 127.491 |
| *Hynobius leechii* | MVZ | 230336 | 1999 | Formalin/EtOH | 37.489 | 127.491 |
| *Hynobius leechii* | MVZ | 230337 | 1999 | Formalin/EtOH | 37.489 | 127.491 |
| *Hynobius leechii* | MVZ | 230338 | 1999 | Formalin/EtOH | 37.489 | 127.491 |
| *Hynobius leechii* | MVZ | 230967 | 1999 | Formalin/EtOH | 37.489 | 127.491 |
| *Hynobius leechii* | MVZ | 233460 | 1998 | Formalin/EtOH | 34.61 | 126.58 |
| *Hynobius leechii* | MVZ | 233461 | 1998 | Formalin/EtOH | 34.61 | 126.58 |
| *Hynobius leechii* | MVZ | 233464 | 1995 | Formalin/EtOH | 38.21 | 128.34 |
| *Hynobius leechii* | MVZ | 233465 | 1995 | Formalin/EtOH | 38.21 | 128.34 |
| *Hynobius leechii* | MVZ | 233466 | 1995 | Formalin/EtOH | 38.21 | 128.34 |
| *Hynobius leechii* | MVZ | 233467 | 1995 | Formalin/EtOH | 38.21 | 128.34 |
| *Hynobius leechii* | MVZ | 233468 | 1995 | Formalin/EtOH | 38.21 | 128.34 |
| *Hynobius leechii* | MVZ | 233469 | 1995 | Formalin/EtOH | 38.21 | 128.34 |
| *Hynobius leechii* | MVZ | 233470 | 1995 | Formalin/EtOH | 38.21 | 128.34 |
| *Hynobius leechii* | MVZ | 233471 | 1995 | Formalin/EtOH | 38.21 | 128.34 |
| *Hynobius yangi* | MVZ | 247158 | 1995 | Formalin/EtOH | 35.31666667 | 129.2833333 |
| *Kaloula borealis* | CAS | 87086 | 1952 | Formalin/EtOH | 38.058 | 127.323 |
| *Kaloula borealis* | CAS | 87087 | 1952 | Formalin/EtOH | 38.058 | 127.323 |
| *Kaloula borealis* | MVZ | 61821 | 1954 | Formalin/EtOH | 37.6 | 126.96 |
| *Kaloula borealis* | MVZ | 66637 | 1954 | Formalin/EtOH | 37.5 | 128 |
| *Karsenia koreana* | MVZ | 246033 | 2004 | Formalin/EtOH | 35.975 | 127.2736 |
| *Karsenia koreana* | MVZ | 247154 | n/a | Formalin/EtOH | n/a | n/a |
| *Karsenia koreana* | MVZ | 247155 | n/a | Formalin/EtOH | n/a | n/a |
| *Karsenia koreana* | MVZ | 247156 | n/a | Formalin/EtOH | n/a | n/a |
| *Karsenia koreana* | MVZ | 247157 | n/a | Formalin/EtOH | n/a | n/a |
| *Onychodactylus fischeri* | MVZ | 173513 | 1979 | Formalin/EtOH | 37.39557 | 128.68037 |
| *Onychodactylus fischeri* | MVZ | 233472 | 1998 | Formalin/EtOH | 37.61 | 127.71 |
| *Onychodactylus fischeri* | MVZ | 233473 | 1998 | Formalin/EtOH | 37.61 | 127.71 |
| *Onychodactylus fischeri* | MVZ | 233474 | 1998 | Formalin/EtOH | 37.61 | 127.71 |
| *Onychodactylus fischeri* | MVZ | 233475 | 1998 | Formalin/EtOH | 37.61 | 127.71 |
| *Onychodactylus fischeri* | MVZ | 233476 | 1998 | Formalin/EtOH | 35.45 | 126.87 |
| *Pelophylax chosenicus* | CAS | 87080 | 1952 | Formalin/EtOH | 37.83241 | 126.81845 |
| *Pelophylax nigromaculatus* | CAS | 15206 | 1953 | Formalin/EtOH | 37.45 | 126.39 |
| *Pelophylax nigromaculatus* | CAS | 86919 | 1952 | Formalin/EtOH | 37.83241 | 126.81845 |
| *Pelophylax nigromaculatus* | CAS | 86920 | 1952 | Formalin/EtOH | 37.83241 | 126.81845 |
| *Pelophylax nigromaculatus* | CAS | 86921 | 1952 | Formalin/EtOH | 37.83241 | 126.81845 |
| *Pelophylax nigromaculatus* | CAS | 86922 | 1952 | Formalin/EtOH | 37.83241 | 126.81845 |
| *Pelophylax nigromaculatus* | CAS | 86923 | 1952 | Formalin/EtOH | 37.83241 | 126.81845 |
| *Pelophylax nigromaculatus* | CAS | 86924 | 1952 | Formalin/EtOH | 37.83241 | 126.81845 |
| *Pelophylax nigromaculatus* | CAS | 86925 | 1952 | Formalin/EtOH | 37.83241 | 126.81845 |
| *Pelophylax nigromaculatus* | CAS | 86926 | 1952 | Formalin/EtOH | 37.83241 | 126.81845 |
| *Pelophylax nigromaculatus* | CAS | 87084 | 1952 | Formalin/EtOH | 37.889 | 126.767 |
| *Pelophylax nigromaculatus* | MVZ | 61822 | 1954 | Formalin/EtOH | 37.76765 | 127.10457 |
| *Pelophylax nigromaculatus* | MVZ | 66658 | 1954 | Formalin/EtOH | n/a | n/a |
| *Pelophylax nigromaculatus* | MVZ | 66659 | 1954 | Formalin/EtOH | n/a | n/a |
| *Pelophylax nigromaculatus* | MVZ | 178455 | 1982 | Formalin/EtOH | 35.16 | 129.155 |
| *Pelophylax nigromaculatus* | MVZ | 178456 | 1982 | Formalin/EtOH | 35.16 | 129.155 |
| *Rana coreana* | CAS | 87018 | 1952 | Formalin/EtOH | 37.83241 | 126.81845 |
| *Rana coreana* | CAS | 87019 | 1952 | Formalin/EtOH | 37.83241 | 126.81845 |
| *Rana coreana* | CAS | 87020 | 1952 | Formalin/EtOH | 37.83241 | 126.81845 |
| *Rana coreana* | CAS | 87021 | 1952 | Formalin/EtOH | 37.83241 | 126.81845 |
| *Rana coreana* | CAS | 87022 | 1952 | Formalin/EtOH | 37.83241 | 126.81845 |
| *Rana coreana* | CAS | 87023 | 1952 | Formalin/EtOH | 37.83241 | 126.81845 |
| *Rana coreana* | CAS | 87024 | 1952 | Formalin/EtOH | 37.83241 | 126.81845 |
| *Rana coreana* | CAS | 87025 | 1952 | Formalin/EtOH | 37.83241 | 126.81845 |
| *Rana coreana* | CAS | 87026 | 1952 | Formalin/EtOH | 37.83241 | 126.81845 |
| *Rana coreana* | CAS | 87027 | 1952 | Formalin/EtOH | 37.83241 | 126.81845 |
| *Rana coreana* | CAS | 87028 | 1952 | Formalin/EtOH | 37.83241 | 126.81845 |
| *Rana coreana* | CAS | 87029 | 1952 | Formalin/EtOH | 37.83241 | 126.81845 |
| *Rana coreana* | CAS | 87030 | 1952 | Formalin/EtOH | 37.83241 | 126.81845 |
| *Rana coreana* | CAS | 87031 | 1952 | Formalin/EtOH | 37.83241 | 126.81845 |
| *Rana coreana* | CAS | 87032 | 1952 | Formalin/EtOH | 37.83241 | 126.81845 |
| *Rana coreana* | CAS | 87033 | 1952 | Formalin/EtOH | 37.83241 | 126.81845 |
| *Rana coreana* | CAS | 87034 | 1952 | Formalin/EtOH | 37.83241 | 126.81845 |
| *Rana coreana* | CAS | 87035 | 1952 | Formalin/EtOH | 37.83241 | 126.81845 |
| *Rana dybowskii* | MVZ | 178457 | 1982 | Formalin/EtOH | 35.16 | 129.155 |
| *Rana dybowskii* | MVZ | 178458 | 1982 | Formalin/EtOH | 35.16 | 129.155 |
| *Rana dybowskii* | MVZ | 178459 | 1982 | Formalin/EtOH | 35.16 | 129.155 |
| *Rana dybowskii* | MVZ | 178460 | 1982 | Formalin/EtOH | 35.16 | 129.155 |
| *Rana dybowskii* | MVZ | 178461 | 1982 | Formalin/EtOH | 35.16 | 129.155 |
| *Rana* sp. | MVZ | 61823 | 1954 | Formalin/EtOH | 37.76765 | 127.10457 |
| *Rana* sp. | MVZ | 61824 | 1954 | Formalin/EtOH | 37.76765 | 127.10457 |
| *Rana* sp. | MVZ | 61825 | 1954 | Formalin/EtOH | 37.76765 | 127.10457 |
| *Rana* sp. | MVZ | 66644 | 1954 | Formalin/EtOH | n/a | n/a |
| *Rana* sp. | MVZ | 66645 | 1954 | Formalin/EtOH | 37.887 | 127.733 |
| *Rana* sp. | MVZ | 66646 | 1954 | Formalin/EtOH | 37.887 | 127.733 |
| *Rana* sp. | MVZ | 66647 | 1954 | Formalin/EtOH | 37.887 | 127.733 |
| *Rana* sp. | MVZ | 66648 | 1954 | Formalin/EtOH | 37.887 | 127.733 |
| *Rana* sp. | MVZ | 66649 | 1954 | Formalin/EtOH | 37.887 | 127.733 |
| *Rana* sp. | MVZ | 66650 | 1954 | Formalin/EtOH | 37.887 | 127.733 |
| *Rana* sp. | MVZ | 66651 | 1954 | Formalin/EtOH | 37.887 | 127.733 |
| *Rana* sp. | MVZ | 66652 | 1954 | Formalin/EtOH | 37.887 | 127.733 |
| *Rana* sp. | MVZ | 66653 | 1954 | Formalin/EtOH | 37.887 | 127.733 |
| *Rana* sp. | MVZ | 66654 | 1954 | Formalin/EtOH | 37.887 | 127.733 |
| *Rana* sp. | MVZ | 66655 | 1954 | Formalin/EtOH | 37.887 | 127.733 |
| *Rana* sp. | MVZ | 66656 | 1954 | Formalin/EtOH | 37.887 | 127.733 |
| *Rana* sp. | MVZ | 66657 | 1954 | Formalin/EtOH | 37.887 | 127.733 |
| *Rana* sp. | MVZ | 66660 | 1954 | Formalin/EtOH | 37.887 | 127.733 |
| *Rana* sp. | MVZ | 66661 | 1954 | Formalin/EtOH | 37.887 | 127.733 |
| *Rana* sp. | MVZ | 66662 | 1954 | Formalin/EtOH | 37.887 | 127.733 |
| *Rana* sp. | MVZ | 66663 | 1954 | Formalin/EtOH | 37.887 | 127.733 |
| *Rana* sp. | MVZ | 66664 | 1954 | Formalin/EtOH | 37.887 | 127.733 |
| *Rana* sp. | MVZ | 66665 | 1954 | Formalin/EtOH | 37.887 | 127.733 |
| *Rana* sp. | MVZ | 66666 | 1954 | Formalin/EtOH | 37.887 | 127.733 |
| *Rana* sp. | MVZ | 66667 | 1954 | Formalin/EtOH | 37.887 | 127.733 |
| *Rana* sp. | MVZ | 66668 | 1954 | Formalin/EtOH | 37.887 | 127.733 |
| *Rana* sp. | MVZ | 66669 | 1954 | Formalin/EtOH | 37.887 | 127.733 |
| *Rana* sp. | MVZ | 66670 | 1954 | Formalin/EtOH | 37.887 | 127.733 |
| *Rana* sp. | MVZ | 66671 | 1954 | Formalin/EtOH | 37.887 | 127.733 |
| *Rana* sp. | MVZ | 66672 | 1954 | Formalin/EtOH | 37.887 | 127.733 |
| *Rana* sp. | MVZ | 66673 | 1954 | Formalin/EtOH | 37.887 | 127.733 |
| *Rana* sp. | MVZ | 66674 | 1954 | Formalin/EtOH | 37.887 | 127.733 |
| *Rana* sp. | MVZ | 66675 | 1954 | Formalin/EtOH | 37.887 | 127.733 |
| *Rugosa emeljanovi* | CAS | 32672 | 1911 | EtOH/EtOH | 39.143 | 127.301 |
| *Rugosa emeljanovi* | CAS | 32673 | 1911 | EtOH/EtOH | 39.143 | 127.301 |
| *Rugosa emeljanovi* | CAS | 32674 | 1911 | EtOH/EtOH | 39.143 | 127.301 |
| *Rugosa emeljanovi* | CAS | 32675 | 1911 | EtOH/EtOH | 39.143 | 127.301 |
| *Rugosa emeljanovi* | CAS | 32676 | 1911 | EtOH/EtOH | 39.143 | 127.301 |
| *Rugosa emeljanovi* | CAS | 32677 | 1911 | EtOH/EtOH | 39.143 | 127.301 |
| *Rugosa emeljanovi* | CAS | 32678 | 1911 | EtOH/EtOH | 39.143 | 127.301 |
| *Rugosa emeljanovi* | CAS | 86936 | 1952 | Formalin/EtOH | 37.83241 | 126.81845 |
| *Rugosa emeljanovi* | CAS | 86937 | 1952 | Formalin/EtOH | 37.83241 | 126.81845 |
| *Rugosa emeljanovi* | CAS | 86938 | 1952 | Formalin/EtOH | 37.83241 | 126.81845 |
| *Rugosa emeljanovi* | CAS | 86939 | 1952 | Formalin/EtOH | 37.83241 | 126.81845 |
| *Rugosa emeljanovi* | CAS | 86940 | 1952 | Formalin/EtOH | 37.83241 | 126.81845 |
| *Rugosa emeljanovi* | CAS | 86941 | 1952 | Formalin/EtOH | 37.83241 | 126.81845 |
| *Rugosa emeljanovi* | CAS | 86942 | 1952 | Formalin/EtOH | 37.83241 | 126.81845 |
| *Rugosa emeljanovi* | CAS | 86943 | 1952 | Formalin/EtOH | 37.83241 | 126.81845 |
| *Rugosa emeljanovi* | CAS | 86944 | 1952 | Formalin/EtOH | 37.83241 | 126.81845 |
| *Rugosa emeljanovi* | CAS | 86945 | 1952 | Formalin/EtOH | 37.83241 | 126.81845 |
| *Rugosa emeljanovi* | CAS | 86946 | 1952 | Formalin/EtOH | 37.83241 | 126.81845 |
| *Rugosa emeljanovi* | CAS | 86947 | 1952 | Formalin/EtOH | 37.83241 | 126.81845 |
| *Rugosa emeljanovi* | CAS | 86948 | 1952 | Formalin/EtOH | 37.83241 | 126.81845 |
| *Rugosa emeljanovi* | CAS | 86949 | 1952 | Formalin/EtOH | 37.83241 | 126.81845 |
| *Rugosa emeljanovi* | CAS | 86950 | 1952 | Formalin/EtOH | 37.83241 | 126.81845 |
| *Rugosa emeljanovi* | CAS | 86951 | 1952 | Formalin/EtOH | 37.83241 | 126.81845 |
| *Rugosa emeljanovi* | CAS | 86952 | 1952 | Formalin/EtOH | 37.83241 | 126.81845 |
| *Rugosa emeljanovi* | CAS | 86953 | 1952 | Formalin/EtOH | 37.83241 | 126.81845 |
| *Rugosa emeljanovi* | CAS | 86954 | 1952 | Formalin/EtOH | 37.83241 | 126.81845 |
| *Rugosa emeljanovi* | CAS | 86955 | 1952 | Formalin/EtOH | 37.83241 | 126.81845 |
| *Rugosa emeljanovi* | CAS | 86956 | 1952 | Formalin/EtOH | 37.83241 | 126.81845 |
| *Rugosa emeljanovi* | CAS | 86957 | 1952 | Formalin/EtOH | 37.83241 | 126.81845 |
| *Rugosa emeljanovi* | CAS | 86958 | 1952 | Formalin/EtOH | 37.83241 | 126.81845 |
| *Rugosa emeljanovi* | CAS | 86959 | 1952 | Formalin/EtOH | 37.83241 | 126.81845 |
| *Rugosa emeljanovi* | CAS | 86960 | 1952 | Formalin/EtOH | 37.83241 | 126.81845 |
| *Rugosa emeljanovi* | CAS | 86961 | 1952 | Formalin/EtOH | 37.83241 | 126.81845 |
| *Rugosa emeljanovi* | CAS | 86962 | 1952 | Formalin/EtOH | 37.83241 | 126.81845 |
| *Rugosa emeljanovi* | CAS | 86963 | 1952 | Formalin/EtOH | 37.83241 | 126.81845 |
| *Rugosa emeljanovi* | CAS | 86964 | 1952 | Formalin/EtOH | 37.83241 | 126.81845 |
| *Rugosa emeljanovi* | CAS | 86965 | 1952 | Formalin/EtOH | 37.83241 | 126.81845 |
| *Rugosa emeljanovi* | CAS | 86966 | 1952 | Formalin/EtOH | 37.83241 | 126.81845 |
| *Rugosa emeljanovi* | CAS | 86967 | 1952 | Formalin/EtOH | 37.83241 | 126.81845 |
| *Rugosa emeljanovi* | CAS | 86968 | 1952 | Formalin/EtOH | 37.83241 | 126.81845 |
| *Rugosa emeljanovi* | CAS | 86969 | 1952 | Formalin/EtOH | 37.83241 | 126.81845 |
| *Rugosa emeljanovi* | CAS | 86970 | 1952 | Formalin/EtOH | 37.83241 | 126.81845 |
| *Rugosa emeljanovi* | CAS | 86971 | 1952 | Formalin/EtOH | 37.83241 | 126.81845 |
| *Rugosa emeljanovi* | CAS | 86972 | 1952 | Formalin/EtOH | 37.83241 | 126.81845 |
| *Rugosa emeljanovi* | CAS | 86973 | 1952 | Formalin/EtOH | 37.83241 | 126.81845 |
| *Rugosa emeljanovi* | CAS | 86974 | 1952 | Formalin/EtOH | 37.83241 | 126.81845 |
| *Rugosa emeljanovi* | CAS | 86975 | 1952 | Formalin/EtOH | 37.83241 | 126.81845 |
| *Rugosa emeljanovi* | MVZ | 61826 | 1954 | Formalin/EtOH | 37.76765 | 127.10457 |
| *Rugosa emeljanovi* | MVZ | 66643 | 1954 | Formalin/EtOH | 37.887 | 127.733 |
